# Visualization of proteomics data using R and Bioconductor

**DOI:** 10.1002/pmic.201400392

**Published:** 2015-04-08

**Authors:** Laurent Gatto, Lisa M Breckels, Thomas Naake, Sebastian Gibb

**Affiliations:** 1 Department of Biochemistry, Cambridge Centre for Proteomics, University of CambridgeCambridge, UK; 2 Department of Biochemistry, Computational Proteomics Unit, University of CambridgeCambridge, UK; 3 Department of Anesthesiology and Intensive Care, University Medicine GreifswaldGreifswald, Germany

**Keywords:** Bioconductor, Bioinformatics, Data analysis, Programming, R, Visualization

## Abstract

Data visualization plays a key role in high-throughput biology. It is an essential tool for data exploration allowing to shed light on data structure and patterns of interest. Visualization is also of paramount importance as a form of communicating data to a broad audience. Here, we provided a short overview of the application of the R software to the visualization of proteomics data. We present a summary of R's plotting systems and how they are used to visualize and understand raw and processed MS-based proteomics data.

## 1. Introduction

Visualization plays an essential role in high-throughput biology. It is a means to explore, understand, and communicate data in a way descriptive statistical properties can often hardly compete with. Anscombe's quartet [1] is a well-known dataset composed of four sets of 11 x–y pairs that are characterized by identical traditional descriptive statistics (variances and means of 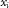 (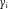) are 11.00 (4.13) and 9.00 (7.50), respectively, and the correlations of 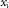 and 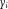 are 0.82, for 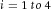) while being strikingly different. While these differences become apparent upon inspection of the regression coefficients, the x–y scatter plots (shown in the RProtVis vignette of the RforProteomics package, see below) illustrate the striking differences between these data and that only the first dataset can be modelled by a linear regression.

The R software is a cross-platform language and environment for statistical computing [2]. It excels in statistical data mining and modeling and possesses very powerful graphics facilities [3] to reveal specific patterns in data. R is an open-source project that has been adopted in various fields in academic research and industry. In this introduction to data visualization using R, we will provide an overview of R's graphical systems and how they are applied to the visualization of proteomics data. A companion vignette complements this work with additional visualizations and provides the detailed code allowing readers to reproduce the figures and experiment with the plotting functions described hereafter. This RProtVis vignette, available in the RforProteomics [4] package, is a dynamically generated document that executes the code chunks and includes their output (text, tables, or figures) in the final document. The vignette also provides general resources about R graphics and visualization. The detailed code is accessed by installing RforProteomics as described on the package landing page (http://bioconductor.org/packages/release/data/experiment/html/RforProteomics.html) and typing:

**Table:** 

library(“RforProteomics”)
## *This is the 'RforProteomics' version 1.3.2*.
##
## *To get started, visit*
## http://lgatto.github.com/RforProteomics/
##
## *or, in R, open package vignettes by typing*
## *RforProteomics() # R/Bioc for proteomics overview*
## *RProtVis() # R/Bioc for proteomics visualization*

There are thousands of add-on *packages* on the Comprehensive R Archive Network (CRAN) servers, openly and freely contributed by developers worldwide. These packages can be easily installed and used to complement R's out-of-the-box functionality; they offer novel statistical functionality, extended support visualization (including interactive visualization) as well as infrastructure dedicated to the analysis and visualization of biological data. The Bioconductor project [5] is an open source and open development initiative that offers over 900 packages for the analysis and comprehension of high-throughput biology data. The Bioconductor packages, as opposed to packages on any other repository, form an environment of interoperable software that rely on each ether's strength. Originally developed for genomics data, the project is benefiting from increasing interest and contribution from the mass spectrometry and proteomics communities; the number of MS and proteomics packages and their number of downloads have doubled in the last 2 years [6]. We redirect readers to Gatto et al. [7] and the RforProteomics vignette [4] for an introduction to R and Bioconductor for the analysis of proteomics data. In this manuscript, we will focus on the visualization capabilities of R itself (Section 3 and R add-on packages and their application to MS and proteomics data (Section 4).

R is widely used in data-driven sciences, and biology in particular. The Bioconductor project [5] has become a vital software platform for the worldwide high-throughput biology research community. Application of these software in MS and proteomics are multiple. Horn et al. [8] have used the Bioconductor package RNAinteract to perform high-throughput identification of genetic interactions in tissue-culture cells through RNAi. They provide detailed documentation of their data and analyses in the RNAinteractMAPK package. Perez-Riverol et al. [9] have developed an approach to estimate peptide isoelectric point values using a Support Vector Machine classifier from the caret package [10]. Colinge et al. [11] have performed integrated human kinase network analysis relying on R for all statistical analyses. Rigbolt et al. [12], in their time course study of the proteome and phosphoproteome of human embryonic stem cell, have used R to identify and visualize temporal protein clusters. Chen et al. [13] used the isobar [14] Bioconductor package to analyze their TMT-based quantitative data [15]. Finally, Groen et al. [16] and Nikolovski et al. [17] used the pRoloc package [18] to analyze and visualize their quantitative MS-based spatial proteomics experiments.

## 2. R graphical systems

**Figure 1:**
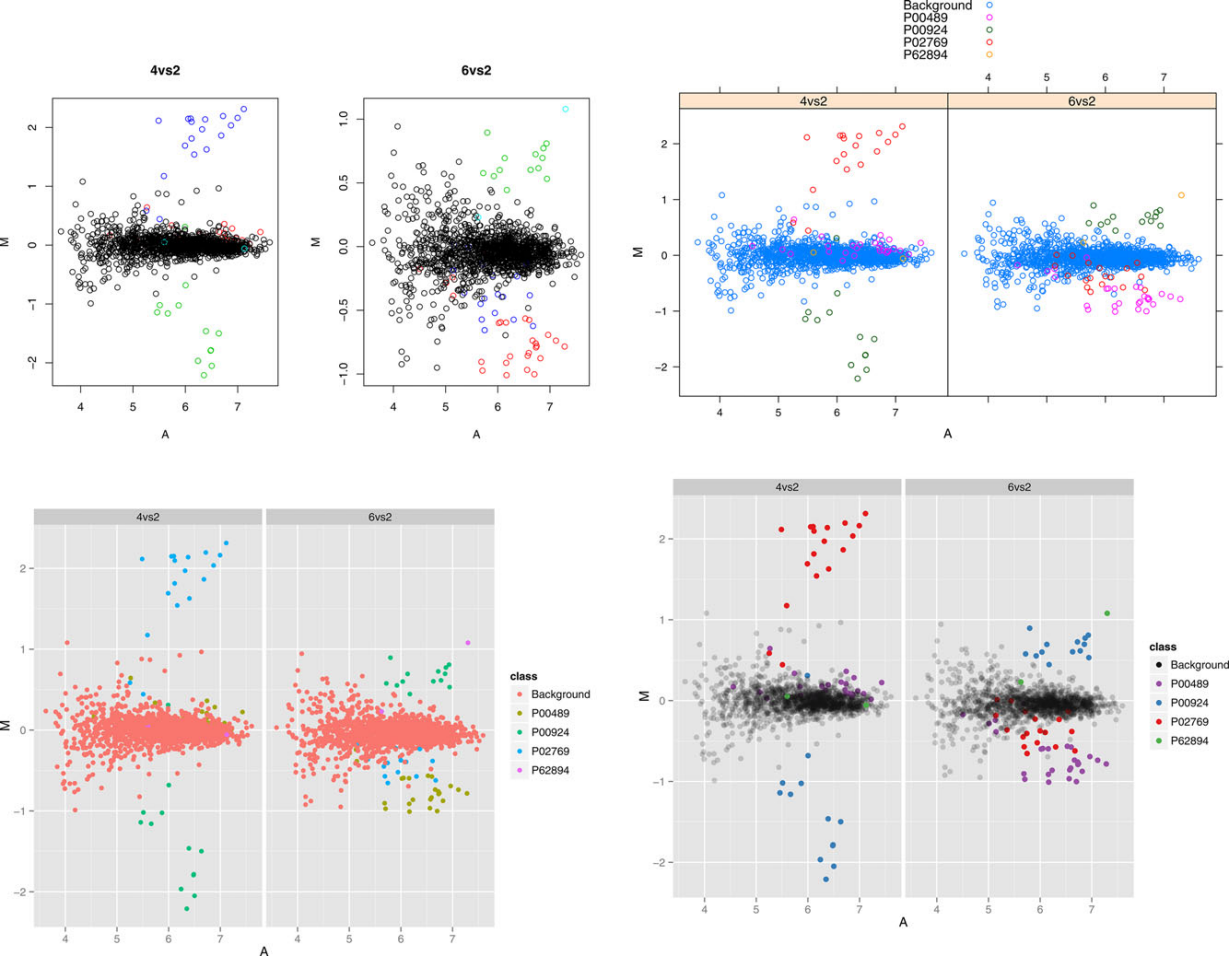
R's plotting systems: traditional system (top left), lattice (top right) and ggplot2 (bottom left) displaying the same data on a MA plot. Coloured points describe one of the four spiked-in proteins and the background proteome. The respective plots have been created with minimal configuration. The customized bottom right representation uses specific colour and transparency contrasts to differentiate background and spiked-in proteins and convey information on the density of points.

There exist two graphics systems in R, namely the traditional graphics facilities and the grid graphics system. The former implements a pen and paper model system where individual graphical items (either graphical primitives such as lines, points, etc. or entire plots) are plotted on top or each other. This plotting infrastructure is fast, with relatively little flexibility. It offers the default, most direct and simple support for plotting. The grid system, on the other hand, offers graphical primitive objects that can be created and manipulated independently of the plot. Most users do not use grid directly to produce complete plots but rather rely on one of the sophisticated, high-level interfaces that easily produce complex figures, namely lattice [19] and ggplot2 [20]. Using grid to implement Cleveland's Trellis system, the lattice package easily produces multipanel conditioning figures. The ggplot2 package implements Wilkinson's Grammar of Graphics [21] to map data to geometric objects (points, lines, etc.) and aesthetic attributes (colour, shape, size, etc.) and optional statistical transformations to produce figures. Figure 1 displays three MA plots produced with the same input data using the three aforementioned systems. MA plots (MA standing originally for microarray), commonly employed in proteomics (e.g. [14,22]) and genomics (e.g. [23–27]), are a convenient pairwise representation of experiments or conditions, displaying the relation between the (average) log_2_ fold-change and the average expression intensity of features between samples or groups. MA plots can be used to illustrate some fundamental requirements of the data, such as the absence of differential expression for most features in the experiment, by verifying that most points centre around the horizontal 0 line. Significantly differentially expressed features tend to be located toward higher absolute log_2_ fold-changes toward the right hand side (i.e large average expressions), while large deviations from 0 for low average expressions are often the result of random fluctuations in the data. The data used for these figures comes from Gatto et al. [7] and is accessible from the ProteomeXchange repository [28] (accession number PXD000001). In this TMT 6-plex experiment [15], four exogenous proteins were spiked into an equimolar *Erwinia carotovora* lysate with varying proportions in each channel of quantitation; yeast enolase (P00924) at 10:5:2.5:1:2.5:10, bovine serum albumin (P02769) at 1:2.5:5:10:5:1, rabbit glycogen phosphorylase (P00489) at 2:2:2:2:1:1 and bovin cytochrome C (P62894) at 1:1:1:1:1:2, processed as described previously and analyzed on an LTQ Orbitrap Velos mass spectrometer (Thermo Scientific). On the figures, we chose to compare channel 4 (TMT 129) and 6 (TMT 131) against 2 (TMT 127), respectively.

The first three MA plots in Fig. 1 have been created using mostly default settings using the traditional plotting system, lattice and ggplot2 and could be customized at leisure to highlight specific attributes of the data. An interesting effect of Trellis plots (the lattice and faceted ggplot2 figures) is the use of a common scale for both parts of the plots (as opposed to the traditional plots that were generated independently), distinctively underlining the differences in log_2_ fold-changes between the two pairwise comparisons. Various visual idioms such as shape, size, colours, grouping, etc. are commonly used to differentiate or annotate data points. Such attributes must be chosen carefully to efficiently convey patterns of interest in the data. For instance, default colors could be improved to highlight the different classes of proteins (spiked-in and background). The RColorBrewer package [29] defines carefully chosen color palettes for thematic maps such as sequential (for ordered data), diverging (divergent continuous data), and qualitative patterns (categorical data). A customized version of the ggplot2 figure, highlighting the different protein classes through qualitative colors and different transparency levels (except for the legend) to communicate information about point density, is presented in the bottom right panel.

These figures were all created using data stored in a generic tabular data.frame. In R, many plotting functions have been designed to operate on input-specific data types (called *classes*) to produce custom visualizations. Using our example data stored in an MSnSet, a data container for quantitative proteomics data defined in the MSnbase package [22], invoking the dedicated MAplot function produces all 15 pairwise MA plots (see the companion vignette for details). The isobar package also defines a specific MA plotting function to visualize data preprocessing and statistically differentially expressed features. Genomics packages such as limma [24] and marray [25] for microarray data analysis, or DESeq2 [26] and edgeR [27] for RNA-Seq data, also offer MA plots for customized out-of-the-box data visualization.

**Figure 2:**
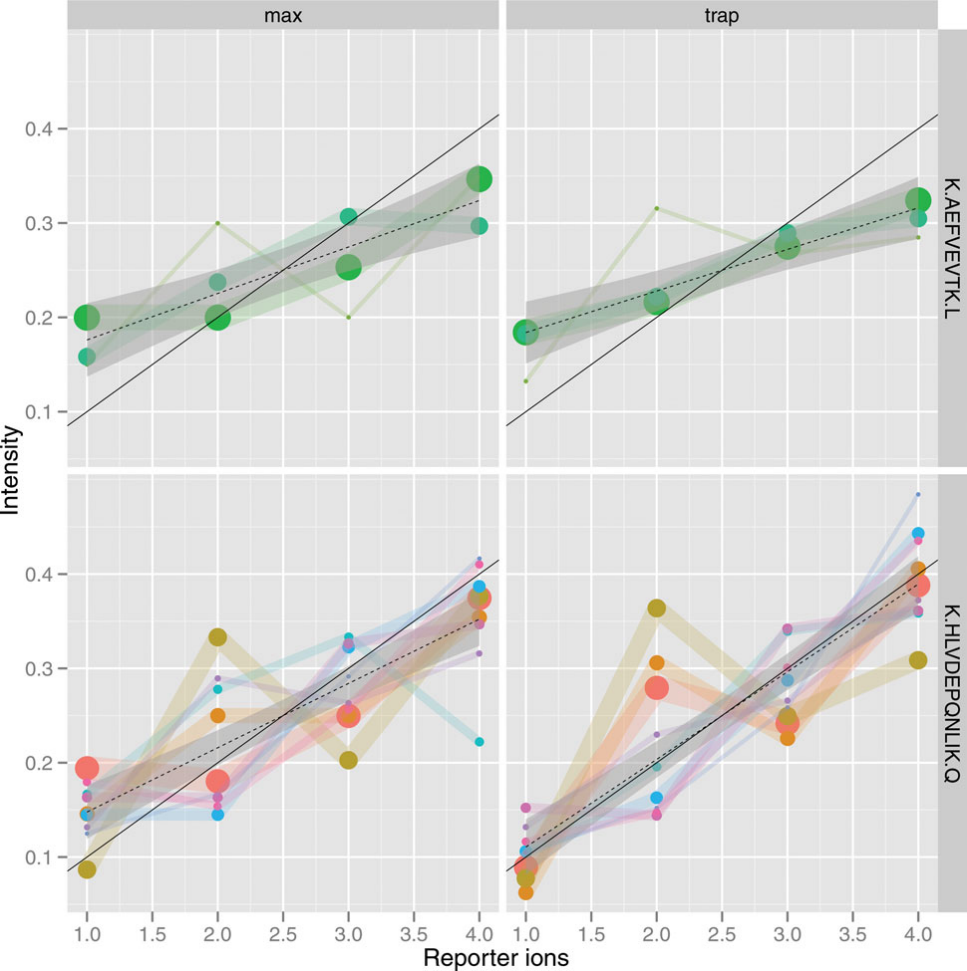
Generating specific visualizations to describe specific properties of the data. Here, we describe the effect of two peak quantitation methods on iTRAQ 4-plex reporter ions for two spiked-in peptides of interest represented by a several peptide spectrum matches each.

Any of these plotting systems can be used to construct specific visualizations to highlight some properties of the data at hand. Often, such figures are created iteratively as part of the data exploration. Figure 2, for example, used the ggplot2 package to compare two ways to estimate peak intensities for an iTRAQ 4-plex experiment [30] along the lattice columns (using the maximum height of the peak or calculating the area under the peak by trapezoidation) and contrasts these two methods for two peptides along the lattice rows. Each peptide is represented by a set of color-coded peptide spectrum match measurements and the size of the points is proportional to the spectrum's total ion count. The solid black line represents the expected relative intensities for these spiked-in features and the dotted line shows the linear regression for the observed data.

Figures can be saved in a variety of formats such as pdf, postscript, png, tiff, svg, etc. which are appropriate for either high quality vector or lightweight bitmap web *static* graphics. Recently, R has been complemented by a range of interactive web-enabled visualization technologies based on modern JavaScript libraries. These frameworks provide new opportunities for data exploration and visualization directly from R, without specific JavaScript, CSS of HTML knowledge. The shinyMA application (https://lgatto.shinyapps.io/shinyMA/) demonstrates a simple shiny [31] application displaying an MA plot and a simple scatter plot with feature-specific expression intensities for two groups (arbitrarily defined as channels 1, 2, 6, and 3, 4, 5, respectively). The two *reactive* plots (see also the screenshot in the accompanying vignette) are linked and are automatically updated upon user-driven mouse events. A click on the MA plots will select its closest point, highlight it with a blue circle and detail the expression values for the selected feature on the scatter plot. The application can be run from the RforProteomics package with the shinyMA() function and directly accessed online. ggvis [32] is recent data visualization package that combines the declarative expressiveness of ggplot2 to describe figures and browser-based interactive visualization (http://ggvis.rstudio.com/). There are several other routes for embedding JavaScript applications through R documented in the *Web Technologies and Services* CRAN task view (http://cran.r-project.org/web/views/WebTechnologies.html).

## 3. Plotting with R

This section presents a very brief introduction to R's basic plotting functionality. There exists numerous R tutorial that also introduce R's plotting systems and syntax. Good plotting reference material for the traditional plotting system and grid can be found in *R Graphics* by Paul Murrell (https://www.stat.auckland.ac.nz/∼paul/RG2e/) [3]. Deepayan Sarkar's book *Lattice: Multivariate Data Visualization with R* [19] provides an in-depth and exhaustive reference to lattice (http://lattice.r-forge.r-project.org/). The ggplot2 book by Hadley Wickham [20] describes theoretical principles of the package but is slightly outdated in terms of syntax; the on-line documentation (http://docs.ggplot2.org/) provides the latest reference. Finally, the *Graphs* chapter in the on-line *Cookbook for R* (http://www.cookbook-r.com/) by Winston Chang or his *R Graphics Cookbook* [33] are excellent practical guides for plotting with R.

*Scatter plots*, i.e. the representation of a set of (*x*, *y*) coordinates in a 2D graph can easily be produced with the plot function. This is the function that was used to create the top left MA plots in figure 1. This function can also be used to produce *volcano plots*, by plotting the log_2_ fold-changes of proteins against 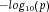 (where *p* is the *p*-value or, better, the *p*-value after adjustment for multiple comparisons) to highlight proteins with large effect size (toward to sides of the graph) and small adjusted *p*-values (toward the top corners of the graph). Some packages offer customized functionality: limma's volcanoplot function for microarray data or msmsTests's [34] res.volcanoplot and MSstats's' [35] groupComparisonPlots (with type VolcanoPlot) for quantitative proteomics data (see examples in the companion vignette).

The Bioconductor package PAA [36] that focuses on the analysis of protein microarray data provides its dedicated plotMAplots and volcanoPlot functions. The plot function can also be used to produce PCA plots, either by manually performing the dimensionality reduction and using the two first principal coordinates to generate the figure with plot, or by using predefined functions such as DESeq2's plotPCA, msmsEDA's [37] count.pca (see the vignette for an example), or pRoloc's plot2D (detailed in Section 4.5. The FactoMineR package [38] also provide functionality data exploration using, among others, PCA and multidimensional scaling (MDS) plots.

Distributions of data points can easily be visualized using *histograms* and *box plots* and variations thereof. Given a vector of values, the hist will produce the corresponding histogram. Continuous histograms, called density plots, can easily be constructed by first computing kernel density estimates using the density function and then plotting these densities using plot. Box plots (also called box-and-whisker plots) can be produced with the boxplot and *violin plots*, which combine box plots and kernel density plots with vioplot function, from the identically named package [39]. Box plots are often used to compare protein or peptide expression distributions in different MS runs (see e.g. the MSstats vignette) or assess the impact of data normalization (see e.g. the MSnbase vignette).

The plot, and more generally matplot functions can also be used to draw *line plots* (instead of the default scatter plots), by specifying that *lines* ought to be used instead of points. These are very useful to represent time course protein expression data or clustering results. An example of the latter can be seen in Fig. 8(detailed in Section 4.5), where the profiles of specific protein clusters of interest are depicted using line plots, or as illustrated in Fig. 7 of Rigbolt et al. [12] where the time course profiles of ten clusters generated by the fuzzy c-mean algorithm produced with Mfuzz package [40] are displayed.

*Bar plots* can easily be produced with the barplot function to illustrate, for example, group sizes. While it is possible to generate *pie charts* with the pie function, these charts are highly undesirable and their use is strongly discouraged. It is indeed difficult to compare different sections of a given pie chart, let alone compare sectors across different pie charts, and they should be replaced by bar plots or *dot plots*, which are easily produced with the dotchart function.

*Heat maps* can easily be produced with the image function. *Dendrograms*, i.e. the visualization of hierarchical clustering, can be produced with the hclust function and visualized with the specific plot method. The dendextend package [41] offers a set of extensions for visualizing and comparing dendrograms. It allows, for example, to easily adjust a tree's graphical parameters such as the color, size, type, etc. of branches, nodes, and labels. Heat maps and dendrograms can easily be combined with the heatmap function. More advanced heat maps can also be produced with the heatmap.2 function from the gplots package [42] and the Bioconductor Heatplus package [43]. The msmsEDA package has specialized counts.heatmap and counts.hc functions that use heatmap.2 and hclust functions to display annotated heat maps and dendrograms for quantitative proteomics data in the MSnSet format. Protein microarray data analyzed with the PAA package can be visualized as heatmaps with the plotFeaturesHeatmap function. Matrix layouts such as heat maps can also be used to graphically represent correlation matrices (see e.g. Figs. 1 in [44] or [9]) using the corrplot package [45].

*Venn diagrams* are the default visualization with which to represent overlaps in, for example, sets of protein identifiers. The limma Bioconductor package provides the vennDiagram function for plotting simple Venn diagrams. The VennDiagram package [46] implements functionality to generate high-resolution Venn and Euler plots and enables various customization.

**Figure 3:**
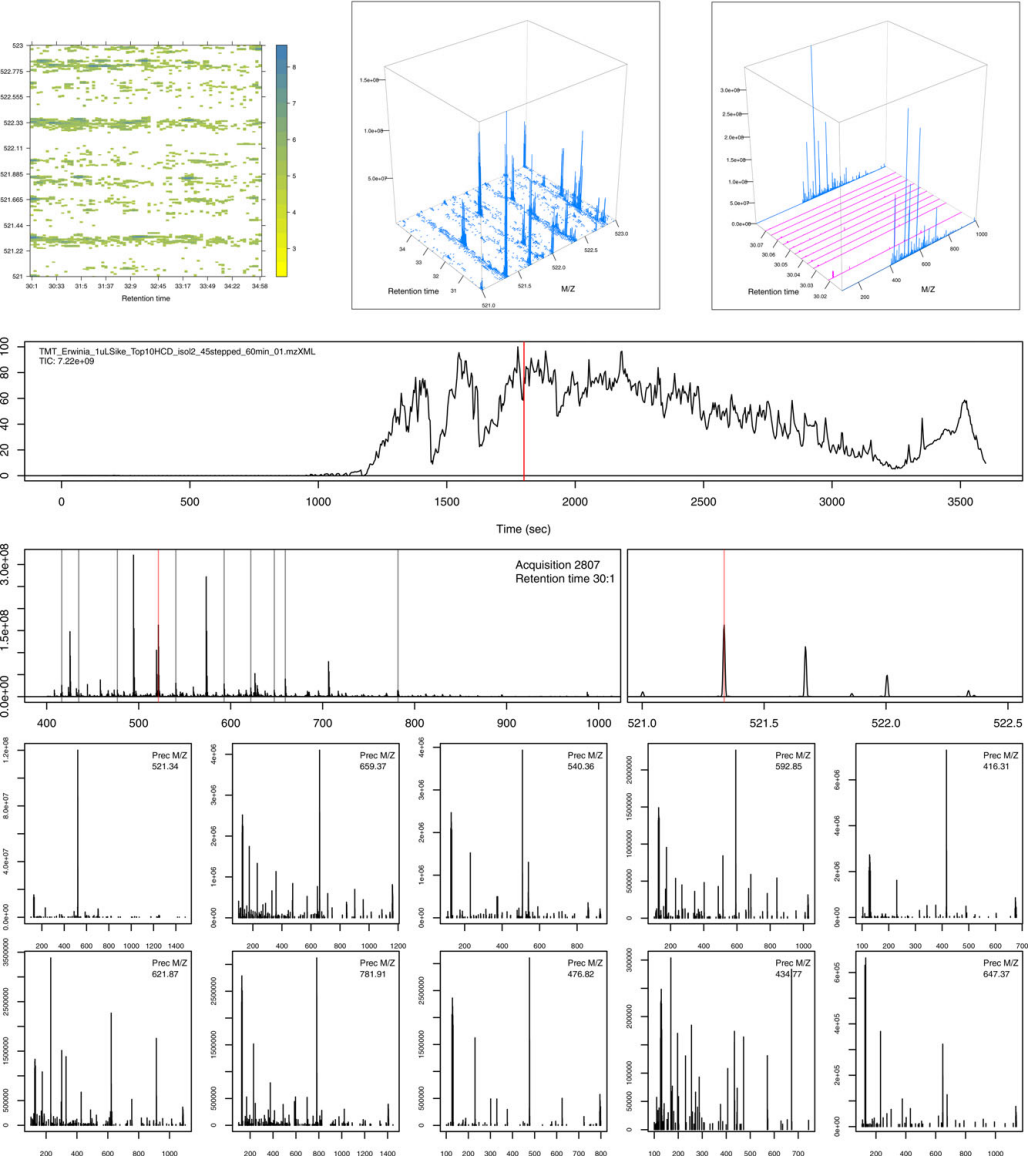
Visualization of raw mass spectrometry data. See text for details. The top figures use the lattice package while the rest have been produced using the traditional graphics infrastructure.

Several packages, such as Rgraphviz [47], graph [48], RBGL [49], and igraph [50], also allow the user to manipulate, perform computations on and plot graphs. It is also possible to produce 3D figures in R using, for example, the rgl package [51], which also allows reorientation of the figure on the interactive plotting display (see e.g. Fig. 3). It is also possible to create animations in R with the animation package [52]. In the vignette, we display animated 3D plots of the raw MS data.

There are of course many more plotting features and graph types that are not described here. The detailed documentation of all these functions can be consulted by preceding the function name by a ? in the R terminal, such as ?heatmap to open the heatmap manual. The RProtVis vignette summarizes the functions above, gives their lattice and ggplot2 equivalents and provides illustrative code snippets and figures using the basic plotting functions and package-specific implementations. We also introduce several plotting customization such as adjusting axis limits, plot annotations, etc. in the vignette.

## 4. Proteomics use cases

In the previous section, we have described general plotting functions. The illustrations used quantitative proteomics data and could be applied to any type of data. In the following sections, we will concentrate on MS- and proteomics-specific visualization. We start with an overview of raw MS data and related data processing visualizations (Section 4.1). We then offer some pointers about quality control (Section 4.2), illustrate the visualization of protein sequence (Section 4.3), imaging MS (Section 4.4) and highlight some spatial proteomics (Section 4.5) -specific visualizations. We focus on packages from the Bioconductor and CRAN repository, as these have passed automatic package checking. In the case of Bioconductor, packages undergo a formal review prior to publication, thus offering some guarantees concerning community best practices in terms of scientific software development and R software dissemination standards, which enable reproducibility of the visualizations and analyses on various systems and over time.

It is worth highlighting several aspects of data visualization using R, or any other programming environment. First, the data used for the visualization was accessed programmatically. In addition, the figures themselves were generated programmatically based on dedicated data structures that have been developed to store, manipulate and process MS or proteomics data. It is also important to emphasize that similar figures can be produced for any other feature (spectra, peptides, or proteins) of interest. More importantly, as the figures are a graphical representation of the underlying data, properties of the data can be computed from the objects that are visualized: quantitation of peaks of interest such as reporter ions and quantitative comparison of spectra, class, and cluster membership of quantitative feature profiles, to mention but a few.

### 4.1 MS data

The mzR package is an interface to raw MS data that supports a variety of widely used formats through the proteowizard software [53], such as mzML [54] and mzXML [55]. The package provides the user with direct low-level, on disk access to raw data and meta-data, as demonstrated in Fig. 3. The top figures illustrate a subset of the retention time versus 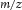 map of the example MS run, using a heat map (left) and 3D representations of the scans over retention time (centre and right). The middle 3D map corresponds exactly to the heat map. The right-most 3D figure shows two MS^1^ scans (blue, acquisition numbers 2807 and 2818) and the interleaved MS^2^ spectra. The first of these MS^1^ spectra, acquisition number 2807, at retention time 30:1 (red bar on the chromatogram on the second row) and subsequent MS^2^ spectra are also depicted on the bottom half of the figures. The middle panel shows the MS^1^ spectrum 2807; the selected ions for MS^2^ analysis are marked by vertical bars. The right-hand side zooms in on a specific precursor ion of 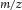 521.34 to show its isotopic envelope. The two last rows present the MS^2^ of the selected ions; these are the same ones as those plotted in purple on the top right 3D map.

**Figure 4:**
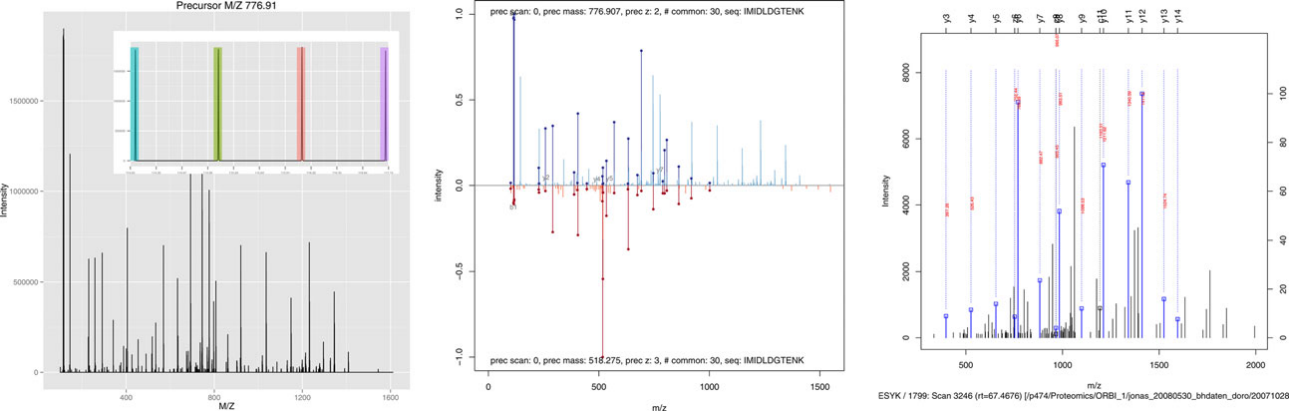
Visualizing MS^2^ spectra using MSnbase (left and centre) and protViz (right). The left figure details the raw data of a spectrum of interest and the iTRAQ 4-plex reporter ions (in profile mode). The central panel compares the centroided spectrum shown on the left (blue, top) with another acquisition of the same peptides (red, bottom). Shared peaks (in bold) and diagnostic *y* and *b* fragments are highlighted. The spectrum on the right has been produced with protViz's peakplot function.

While it is sometimes essential to gain access to low-level data, other packages provide convenient data abstractions to facilitate data manipulation and processing. MSnbase [22] provides such an interface for raw MS^2^-level data, termed MSnExp (Fig. 4, left panel, illustrates an iTRAQ 4-plex MS^2^ spectrum) and quantitative data for any quantitation pipeline, termed MSnSet (see the MAplot function above and Section 4.5 for visualization examples). Data abstraction enables the combination of information from different sources while maintaining simple and coherent interfaces. The central panel in Fig. 4 compares two MS^2^ spectra of the same peptide (IMIDLDGTENK), of charge states 2 and 3, respectively. The protViz package [56] also provides functionality to plot and label peptide fragment mass spectra, as shown on the right panel of Fig. 4.

**Figure 5:**
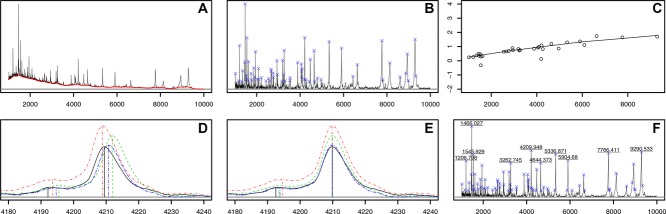
Illustration of the MALDIquant preprocessing pipeline. The first figure (A) shows a raw spectrum with the estimated baseline. In the second figure (B) the spectrum is variance-stabilized, smoothed, baseline-corrected and the detected peaks are marked with blue crosses. The third figure (C) is an example of a fitted warping function for peak alignment. In the next figures, four peaks are shown before (D) and after (E) performing the alignment. The last figure (F) represents a merged spectrum with discovered and labelled peaks.

MALDIquant [57] is a framework for the processing and analysis of MALDI-TOF and other 2D MS^1^-level data. Its plotting functions are based on the traditional graphics infrastructure. It is very fast and flexible and allows the user to investigate each single preprocessing step in detail, as illustrated in Fig. 5. Another important Bioconductor package that provides access and processing support for MS data and related visualization functionality is xcms [58]. It is widely used in the metabolomics community and enables LC/MS data preprocessing for relative quantitation and statistical analysis and enables visualization throughout the pipeline. Other packages provide functionality to process raw MS data, such as, among others MassSpecWavelet [59] continuous wavelet transform for peak detection (used internally by xcms), BRAIN [60] to to calculate the aggregated isotopic distribution, PROcess [61] for SELDI-TOF data processing. The readers are referred to the respective documentation and vignettes for more details.

### 4.2 Quality control

Quality control is often a visualization-intensive task, where specific metrics of interest are plotted for either multiple samples in an experiment, all features in a single experiment or several MS runs are plotted to highlight systematic outliers or dubious patterns. These quality plots often apply basic plotting functions, such as those briefly introduced in Section 3, or their lattice and ggplot2 equivalents. For example, the plotMzDelta function from the MSnbase package implements the histogram of mass delta distributions described in Foster et al. [44] (Fig. 4). The function takes an MSnExp object, that contains all MS^2^ spectra of an MS acquisition, calculates the differences between the top 10% peaks of the MS^2^ and produces the corresponding histogram (see the 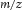 delta plot in the MSnbase-demo vignette). A typical experiment of good quality will display high 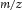 values primarily representing amino acid residue masses. In case of PEG contamination, the histogram will display a high bar around 44 Da (the PEG monomer) at the expense of the actual peptide fragmentation products. The proteoQC Bioconductor package [62] automatically generates an interactive report displaying various quality metrics using bar plots, histograms, density plots, box plots, and Venn diagrams, as described in Section 3.

### 4.3 Genomic and protein sequences

In addition to experimental measurements, visualization can also be applied to annotation and meta-data to highlight the general context of experiments, such as sequence data and genomic context as described below.

The approach taken by ggplot2 where data, instead of being directly represented by graphical primitives, are mapped to geometrical objects and aesthetics attributes has also been applied to high-throughout biological data by ggbio [63]. The package leverages the statistical functionality of R, the data management capabilities of the Bioconductor infrastructure and the concepts of the grammar of graphics to provide high level and modular views of genomic regions, sequence alignments, or splicing patterns.

**Figure 6:**
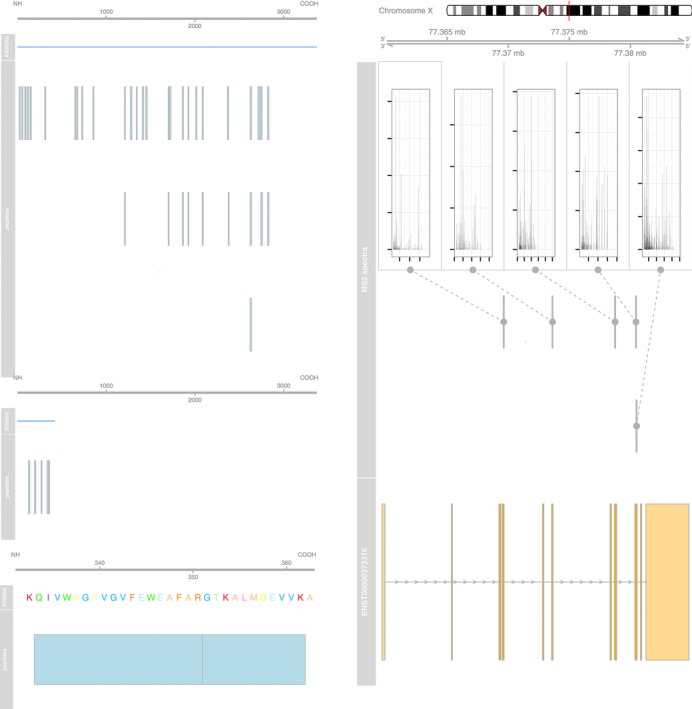
Visualization of proteins and peptides data. The left figures represent peptides along their protein coordinates. On the right, peptides are mapped along genomic coordinates along the underlying gene model.

Genome browsers are popular tools with which to visualize genomic data along a variety of genomic features such as gene or transcript models and other quantitative of qualitative properties. Such tracks can be fetched from public repositories such as ENSEMBL or UCSC, or produced from specific experimental data or custom annotation. The Gviz package [64], and its extension for proteomics data Pviz [65], apply these principles within the Bioconductor framework. Tracks of genomic regions and annotations are created individually, combined and plotted together along a shared coordinate systems. Figure 6 illustrates the application of the latter packages to proteomics data in the frame of the Pbase infrastructure [66]. These figures were constructed from protein sequences (typically the fasta file that was used to identify peptide spectrum matches) and identification data (typically the corresponding mzIdentML file [67]). The top left panel shows two of such proteins, A4UGR9 and P00558 (top tracks, blue lines), with their identified peptides (second tracks). On the bottom left panel, the region spanning from the 331 to the 363 amino acids of P00558 are plotted; at that level, individual amino acid letters are automatically displayed. The visualization on the right represents the peptides mapped to the Phosphoglycerate kinase 1 protein (P00558) along its coordinates on X chromosome and the ENSEMBL gene model. The peptides are decorated with their spectrum matches, that have been extracted from the raw data.

### 4.4 MS imaging

**Figure 7:**
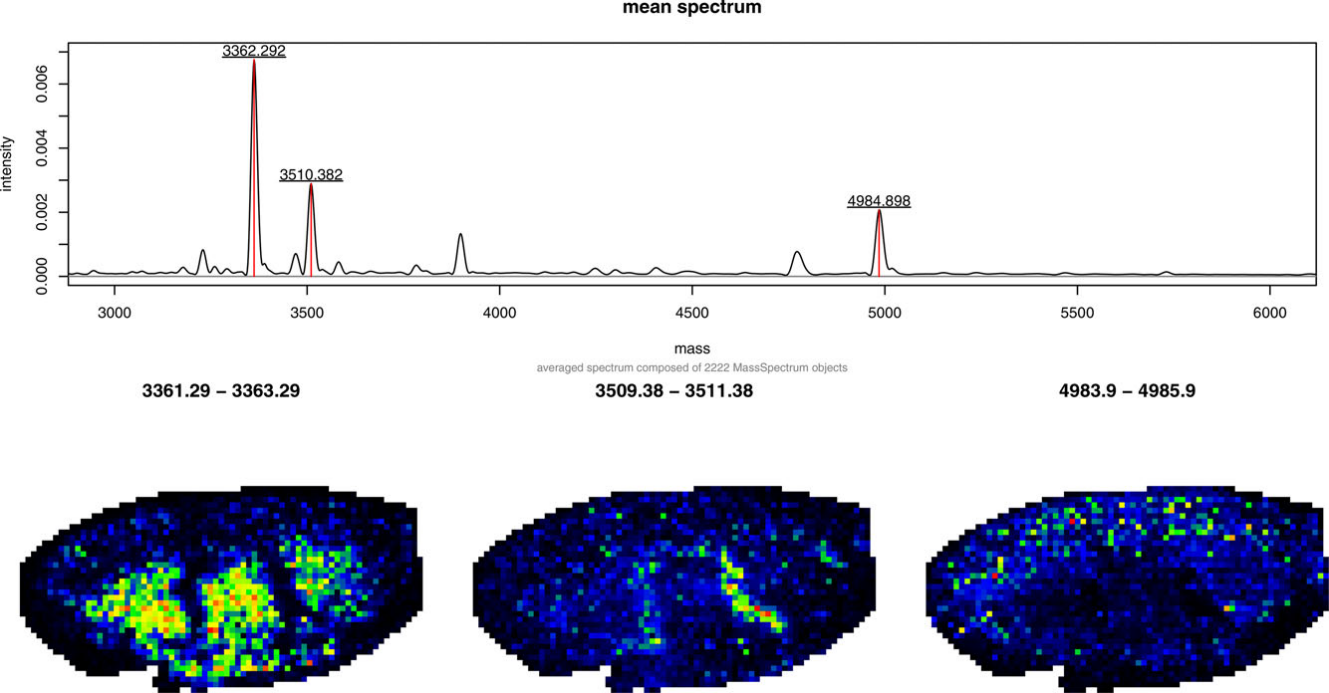
Visualization of MALDI imaging data from a mouse kidney [68]. The top panel shows the mean spectrum for the whole dataset with the three highest peaks labelled. Below three slices covering a 2 Da 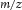 range around the selected peaks are depicted. Each pixel in these slices is the sum of all intensities in the respective 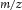 range of the corresponding spectrum. The intensity values are represented by colours (from low to high intensity: black–blue–green–yellow–red).

MS imaging is an emerging technology that allows users to investigate molecular distributions along biological tissues and offers a spatial resolution up to a few micrometres. Generally, a thin tissue section is covered with a MALDI matrix. Ions are then produced by typical MALDI desorption/ionization and spectra are then acquired along a rectangular grid. Eventually, a dataset containing a molecular spectrum profile for each individual pixel (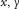 coordinates in the grid) with four dimensions (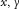, 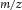, intensity) is produced. While each single spectrum could be processed with the standard preprocessing pipelines mentioned in Section 4.1, there are special needs for the visualization of these datasets. MALDIquant provides functions with which to plot average spectra for specific locations and create “slices” for user-defined 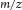 ranges. In Fig. 7, we use a MALDI imaging dataset from a mouse kidney [68] as a demonstration. The dataset is summarized by a mean spectrum and three slices covering a range of 2 Da around the three highest peaks. A recent addition to the Bioconductor project is the Cardinal package [69]; it provides a set of computational tools for MS imaging, such as visualization, preprocessing, and multivariate statistical techniques and supports both MALDI and desorption electrospray ionization-based imaging workflows.

### 4.5 Spatial proteomics

Organelle proteomics, or spatial proteomics, is the systematic study of proteins and their assignment to subcellular niches including organelles. Many methods exist for characterizing the protein complement of organelles, ranging from single-cell proteomic methods that employ microscopy-based techniques, to high-throughput MS-based strategies that assay a population of cells. In this section, we focus on both the importance of visualization and the common methods available for the visualization of high-throughput quantitative proteomics data.

**Figure 8:**
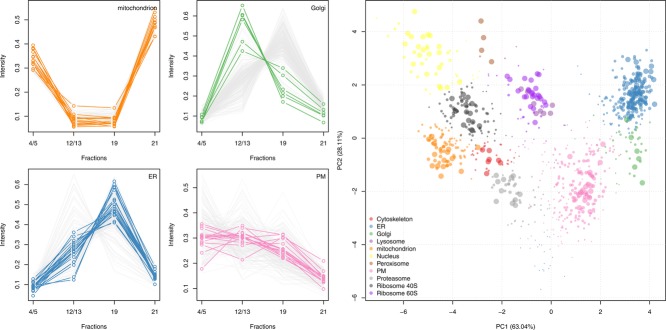
Visualization of spatial proteomics data. Data are from a LOPIT experiment on *Drosophila* embryos [75]. Left: Direct visualization of the protein profiles along the gradient using the plotDist function from the pRoloc package. The profiles are those of the original marker sets from [75] and permits to only show a selection of profiles per plot to be able to distinguish profile trends. Right: A non-linear kernel principal components analysis (kPCA) has been used to reduce the 4 dimensional dataset to 2 dimensions to allow visualization of all proteins on a simple scatter plot through the use of the plot2D function; each vector of 4 points on the protein profile plots has been replaced by a point on the PCA plot. Marker proteins belonging the 11 different subcellular niches are highlighted using different colours and the size of the points have been adapted to reflect the resultant SVM classification scores.

Several high-throughput quantitative methods have been developed that involve the partial separation of organelles across a fractionation scheme coupled with computational analysis. For example, the localization of organelle proteins by isotope tagging (LOPIT) method [70], employs isobaric tagging coupled with MS/MS to capture the distribution of organelle proteins within fractions taken from density gradients. Protein correlation profiling (PCP) is a similar approach that uses label-free quantitation [71]. Both types of gradient-based experiments result in a *m* x *n* matrix of quantitation values wherein the *m* rows represent proteins and the *n* columns contain the relative abundance measurements that approximates the protein distributions along the gradient across the fractions employed (Fig. 8, left). The protein distribution profiles are commonly mapped in-silico to known protein organelle associations (termed marker proteins) via supervised machine learning algorithms (e.g. [72]), creating classifiers that assign unannotated proteins to specific organelles. The underlying principle of gradient-based spatial proteomics is that organelles are separated along the gradient and, as such, one is able to generate organelle-specific distributions along the gradient fractions. We expect proteins belonging to the same organelle to share a similar distribution pattern [73] and therefore cocluster in n-dimensional space.

The most natural and commonly used method with which to visualize and analyze multidimensional data is dimensionality reduction. There are a number of methods that have been developed to project multidimensional data to lower dimensions. Among the most popular, and most widely used in many areas of data analysis are PCA and MDS. PCA transforms the original high dimensional data into a set of linearly uncorrelated variables (principal components) such that the first principal component accounts for as much variability in the data as possible, and that each succeeding component in turn has the highest variance possible under the constraint that it be orthogonal to the preceding components. It is extremely useful for visualizing quantitative spatial proteomics data, firstly to check if there is any underlying structure (i.e. organelle separation) in the data and, secondly, for the identification of any hidden patterns in the data (that may represent organelles and other subcellular niches). R's general prcomp PCA function, part of the stats package, can be specialized for this specific application through use of the plot2D function in the pRoloc package [7], which uses quantitative data in the form of MSnSet instances and implements its visualization using the traditional plotting system. Results from further downstream cluster discovery [74] and organelle classification experiments using machine learning classification methods, e.g. the support vector machine (SVM), are visualized in Fig. 8 (right) for *Drosophila* embryos from Tan et al. [75]. Figure 8 displays 11 subcellular niches and the output SVM scores from classification experiment have been used to set the point size and visualize classification accuracy.

## 5. Conclusion and discussion

In this manuscript, we have described a set of visualizations for MS and proteomics data produced with R and dedicated Bioconductor packages. For a more general introduction to R and Bioconductor applied to the exploration and analysis of MS and proteomics data, readers are invited to consult Gatto et al. [7] and the accompanying RforProteomics vignette. The latter also features numerous relevant visualizations. Indeed, in R, data analysis and visualization are intimately linked. Every Bioconductor package will propose some form of visualization using either generic and dedicated plots to highlight specific patterns in the data or to illustrate the effect of a particular data transformation. At the time of writing, there were 67 and 46 Bioconductor packages annotated with *Proteomics* and *Mass spectrometry* tags (termed *biocViews*), respectively, which can be navigated online (http://bioconductor.org/packages/release/BiocViews.html) or through the RforProteomics package.

Useful visualizations can be produced for every question and every dataset under scrutiny. In some cases, given a specific data type, a simple call to a customized plot method will produce a standard output (e.g. Fig. 4). In other circumstances, specifically customized visualizations are crafted as in Fig. 2. The former are the result of the development of tailored software and infrastructure for specific requirements (MS data, protein sequences, imaging, etc.) and can generally be easily applied out of the box. The latter comes with R's *flexibility* and *expressiveness* and often requires the user to be familiar with R and the graphics system at hand. A more recent set of visualizations that elegantly completes R's capabilities are interactive web-oriented technologies that leverage the flexibility and interactivity of JavaScript. The possibility of combining R's statistical and data mining strengths with the facile development of interactive visualization applications has proven to be an extremely enriching and successful synergy. The shiny web application framework [31] has also spun the offering of various tailored and cross-platform graphical data exploration interfaces throughout the R and Bioconductor communities (see e.g. the interactiveDisplay [76], MSGFgui [77], or pRolocGUI [78] applications).

We have briefly cited some specific data structures tailored toward storing MS and proteomics data, as well as tools with which to produce relevant visualizations. Another important aspect is access to data and, particularly in the light of the programming environment of R, programmatic access to data. The data used to produce the visualizations presented in the review and the accompanying vignette are loaded directly from packages (or specific experimental data packages) into the R environment or downloaded from repositories and imported into R without manual intervention. For example, whole shotgun MS experiments from the PRIDE database can be accessed using the rpx package [79]. Using a unique data identifier such as PXD000001 (see above), or PXD000561, to access the 2384 files from the human draft proteome data from Kim et al. [80], rpx enables the repository for available files to be queried, relevant experiment metadata to be extracted and some or all associated files to be downloaded. Data from the Human Protein Atlas [81,82], the UniProt and PSICQUIC repositories are also accessible from R using the hpar [83], UniProt.ws [84] or biomaRt [85,86], and PSICQUIC [87] packages. Programmatic access to data and metadata offers access to a wide range of resources, including, for example, genomics data such as the ArrayExpress Database at European Bioinformatics Institute (ArrayExpress package [88]), NCBI gene expression omnibus (GEO) (GEOquery package [89]), and sequence read archive (SRA) (SRAdb package [90]) public repositories. Such mechanisms that enable distribution and access the growing number of well-curated and publicly available datasets are critical in facilitating access, and hence reanalysis of data, as well as favour reproducible data analysis.

The Bioconductor project offers a wide selection of packages to obtain biological annotations. In addition to the interfaces to the Human Protein Atlas, UniProt, Biomart, and PSICQUIC resources described above, hundreds of dedicated *annotation* packages distribute functional and organism-specific annotations, offering extensive facilities (http://bioconductor.org/help/workflows/annotation/annotation/) for mapping between microarray probe, genes, and proteins, pathways, gene ontology, homology, etc. These can be used to filter data and annotate plots. One can, for example, retrieve GO [91] annotations for a list of proteins of interest and illustrate the relative enrichments using a bar chart. Such analyses, and more elaborate ones, can of course also be performed using dedicated software packages such as GOstats [92], gage [93], SPIA [94], and many others.

Visualization is an integral part of the data analysis. As soon as data becomes available, whether raw or processed, quantitative of qualitative, there are opportunities for exploration through visualization. Effective visualization goes beyond the display of the data, and must be combined with data filtering, annotation, statistical transformations, aggregation, and summarization, etc. to reveal important patterns. The R software and the Bioconductor infrastructure offer a rich tool set and a flexible environment to achieve these goals. Although an elaborated environment and programming language like R has undeniable strengths, the flexibility, and rich catalog it offers can be intimidating. Another noteworthy obstacle is the command line interface that new users need to familiarize themselves with. The *RStudio* development environment (http://www.rstudio.com/) offers an easy to install and learn interface to the R software; the interaction with R itself is still command line-driven, but a familiar integrated graphical interface, a powerful editor (with tab-completion, syntax highlighting, etc.), a broad community and great support facilitate the dissemination and adoption of R in the experimental life science community. Importantly for our topic of interest, plotting is particularly easy once the first rudiments of the R environment have been acquired. A well formatted spreadsheet can be imported with a single command (using e.g. the read.table function), and will be ready to be plotted using any of the functions described in Section 3.

A major difficulty for newcomers to R is probably the vast amount of functions and packages that are available, which make it unnecessarily difficult to apprehend. Fortunately, there are numerous course, tutorials, and books available, aimed at plain R, as well a specific to specific biological applications (see e.g. the Bioconductor proteomics workflow (http://bioconductor.org/help/workflows/proteomics/)). R and Bioconductor enjoy large communities of users and developers that offer support on public mailing lists and support site (see e.g. https://support.bioconductor.org/).

The programmatic interface to data access, data manipulation, and plotting offers an ideal route to literate programming [95] and reproducible research [96–99]. It enables the user to control every aspect of the analysis, including plotting, and to store these in a source code script that can be reproduced with the same data at a later stage (possibly including further specific customization) or applied to another dataset. Programming a visualization (and implicit or explicit data transformations) enables *transparency* in the way the visualization has been produced, from data acquisition to rendering. This mode of operation enables to dynamically generate reports (using Sweave [100] or knitr [101,102]) that contain the description of the analysis, the calculations and the visualizations. An example of such report generation is described in the qcmetrics package [103] for raw MS data and ^15^N metabolic labeling quality control.

It is important to emphasize that effective visualization is not about mastering a specific tool, programming language or R's many plotting system, but about conferring an enlightening visual message to highlight specific patterns in data. Data visualization is often part of the data exploration and evolves while our understanding of the properties and structure of the data are refined. Data processing, modeling and abstraction, statistical transformations, information density, and levels of detail must often be explored and contrasted to effectively communicate information. It is also advised to start the development of a visualization with a focus on functionality and, at a later stage, optimize for aesthetics to obtain meaningful and visually appealing data representations.

Visualization is an ubiquitous tool in high-throughput disciplines such as genomics and proteomics. Wet-lab scientists, bioinformatics analysts and scientific software developers actively represent their data in numerous ways as means of quality control, data analysis, and interpretation and R is a candidate of choice. Dedicated packages offer unique opportunities for visualization to users without extensive computational background, as well as an appealing route to learning R, while the flexibility and versatility of the environment provides power users with unprecedented means for in-depth exploration.

## Abbreviations

kPCA: kernel PCA
LOPIT: localization of organelle proteins by isotope tagging
MDS: multidimensional scaling
PCP: protein correlation profiling
SVM: support vector machines.
